# Supplementary material for: Triboelectric-Electromagnetic Hybrid Wind-Energy Harvester with a Low Startup Wind Speed in Urban Self-Powered Sensing
Source: Micromachines (Basel). 2023 Jan 23;14(2):298. doi: 10.3390/mi14020298 (PMC9962631; doi:10.3390/mi14020298)
Supplement: Supplementary file 1 [file micromachines-14-00298-s001.zip › micromachines-2180783-highlights - final version.pdf]

- Design for paddles with fluid dynamics in a hybrid energy harvester
- The triboelectric-electromagnetic hybrid harvester activates at wind speeds as low as 1.2 m/s
- Powering a sensor with multiple integrated components in 1.7 m/s wind speeds
- Driving a Bluetooth temperature and humidity sensor in 2.7 m/s wind speeds
