# Supplementary material for: Triboelectric-Electromagnetic Hybrid Wind-Energy Harvester with a Low Startup Wind Speed in Urban Self-Powered Sensing
Source: Micromachines (Basel). 2023 Jan 23;14(2):298. doi: 10.3390/mi14020298 (PMC9962631; doi:10.3390/mi14020298)
Supplement: Supplementary file 1 [file micromachines-14-00298-s001.zip › micromachines-2180783-supplementary - final version.pdf]

micromachines

MDPI

Supporting Information

Triboelectric-Electromagnetic Hybrid Wind-Energy Harvester with a Low Startup Wind Speed in Urban Self-Powered Sensing

Gang Li, Juan Cui *, Tingshan Liu, Yongqiu Zheng, Congcong Hao, Xiaojian Hao and Chenyang Xue

Key Laboratory of Instrumentation Science & Dynamic Measurement, School of Instrument and Electronics, North University of China, Taiyuan 030051, China; s2006024@st.nuc.edu.cn (G.L.); sz202106086@st.nuc.edu.cn (T.L.); zhengyongqiu@nuc.edu.cn (Y.Z.); 20210093@nuc.edu.cn (C.H.); haoxiaojian@nuc.edu.cn (X.H.); xuechenyang@nuc.edu.cn (C.X.)

* Correspondence: cuijuan@nuc.edu.cn

1. Peak Power and Average Power of TENG in LSWS-TEH

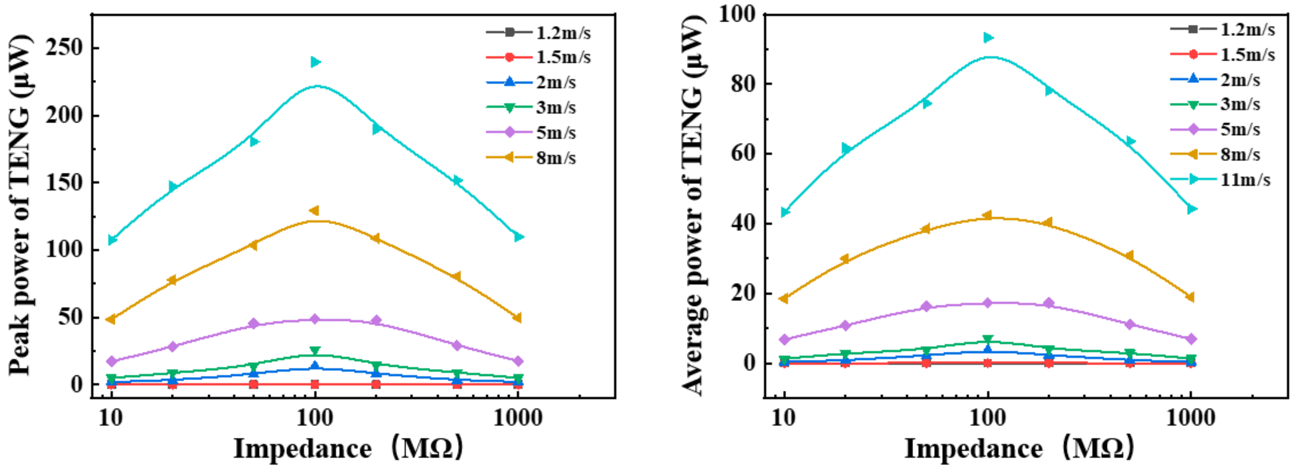

Figure S1. Peak power and average power of TENG in LSWS-TEH.

2. Peak Power and Short-circuit Peak Current of EMG in LSWS-TEH

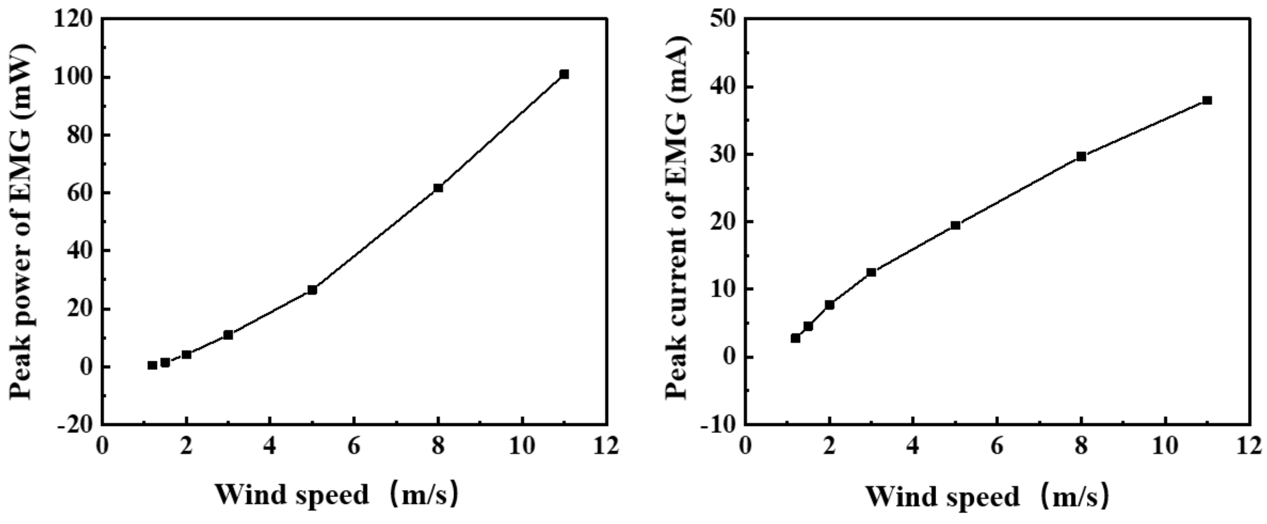

Figure S2. Peak power and short-circuit peak current of EMG in LSWS-TEH.

3. Photo of Switching Regulator Circuit

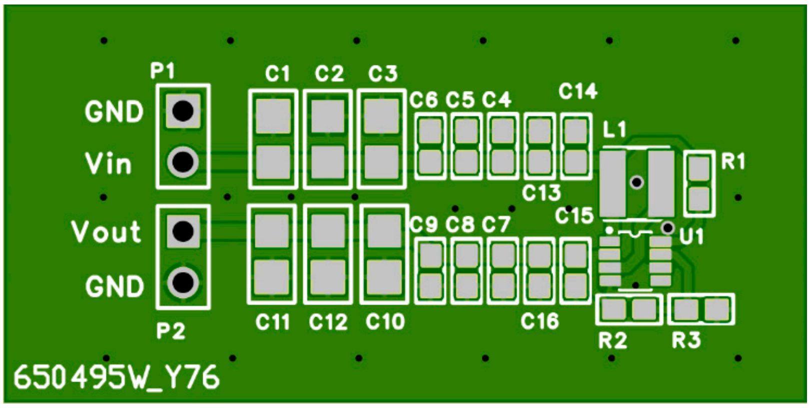

Figure S3. Switching regulator circuit.

4. Comparison Table of Parameters

Table S1. The performance comparison of different triboelectric–electromagnetic hybrid wind-energy harvesters.

References	Size (Including propeller)	Start up wind speeds	Wind speeds for drive sensors	Wind speeds for drive Bluetooth sensors	Operating range	Peak output voltage of TENG/EMG	Peak output current of TENG/EMG	Peak Power generated of TENG/EMG
S1 Nano Energy, 2020, 68: 104319	150 × 100 × 100 mm	4 m/s	-	9 m/s	4–15 m/s	350/60 V @15 m/s	0.007/1.5mA @15 m/s	2.45/64mW @15 m/s
S2 Appl Energ, 2022, 307: 118311	Φ 200 × 170 mm	4 m/s	-	8 m/s	4–21 m/s	390/22 V @9 m/s	0.012/5mA @9 m/s	5.2/14mW @9 m/s
S3 ACS Nano, 2018, 12: 9433–9440	Φ 100 × 160 mm	3.5 m/s	5.7 m/s	-	3.5–9 m/s	65/7.5 V @1000 rpm	–/95mA @1000 rpm	0.54/200mW @1000 rpm
S4 Adv Energy Mater, 2021, 11: 2101194	Φ 180 × 200 mm	3 m/s	-	4.7 m/s	3–15 m/s	7.5/25 V @180/500 rpm	0.0003/12 mA @180/500 rpm	0.002/288 mW @180/500 rpm
**This work**	**Φ 80 × 30 mm**	**1.2 m/s**	**1.7 m/s**	**2.7 m/s**	**1.2–11 m/s**	**84/3.4 V @11 m/s**	**0.005/40mA @11 m/s**	**0.42/100mW @11 m/s**

References

S1. Fan, X.; He, J.; Mu, J.; Qian, J.; Zhang, N.; Yang, C.; Hou, X.; Geng, W.; Wang, X.; Chou, X. Triboelectric-electromagnetic hybrid nanogenerator driven by wind for self-powered wireless transmission in Internet of Things and self-powered wind speed sensor. *Nano Energy* **2020**, *68*, doi:10.1016/j.nanoen.2019.104319.

S2. Li, X.; Gao, Q.; Cao, Y.; Yang, Y.; Liu, S.; Wang, Z.L.; Cheng, T. Optimization strategy of wind energy harvesting via triboelectric-electromagnetic flexible cooperation. *Appl Energ* **2022**, *307*, doi:10.1016/j.apenergy.2021.118311.

S3. Wang, P.; Pan, L.; Wang, J.; Xu, M.; Dai, G.; Zou, H.; Dong, K.; Wang, Z.L. An Ultra-Low-Friction Triboelectric-Electromagnetic Hybrid Nanogenerator for Rotation Energy Harvesting and Self-Powered Wind Speed Sensor. *ACS Nano* **2018**, *12*, 9433-9440, doi:10.1021/acsnano.8b04654.

S4. Yong, S.; Wang, J.; Yang, L.; Wang, H.; Luo, H.; Liao, R.; Wang, Z.L. Auto‐Switching Self‐Powered System for Efficient Broad‐Band Wind Energy Harvesting Based on Dual‐Rotation Shaft Triboelectric Nanogenerator. *Adv Energy Mater* **2021**, *11*, doi:10.1002/aenm.202101194.

Disclaimer/Publisher's Note: The statements, opinions and data contained in all publications are solely those of the individual author(s) and contributor(s) and not of MDPI and/or the editor(s). MDPI and/or the editor(s) disclaim responsibility for any injury to people or property resulting from any ideas, methods, instructions or products referred to in the content.
